# First report of Cassava brown streak viruses on wild plant species in Mozambique^☆^

**DOI:** 10.1016/j.pmpp.2018.10.005

**Published:** 2019-01

**Authors:** Jamisse J.G. Amisse, Joseph Ndunguru, Fred Tairo, Laura M. Boykin, Monica A. Kehoe, Nurbibi Cossa, Elijah Ateka, Chrissie Rey, Peter Sseruwagi

**Affiliations:** a Mozambique Agricultural Research Institute, Posto Agronomico de Nampula, Nampula, Mozambique; b Mikocheni Agricultural Research Institute, Dar es Salaam, Tanzania; c The University of Western Australia, ARC Centre of Excellence in Plant Energy Biology and School of Chemistry and Biochemistry, Crawley, 6009, Western Australia, Australia; d Department of Primary Industries and Regional Development, DPIRD Diagnostic Laboratory Services, South Perth, 6151, Australia; e Mozambique Agricultural Research Institute, Maputo, 2698, Mozambique; f Department of Horticulture, Jomo Kenyatta University of Agriculture and Technology, Nairobi, Kenya; g University of the Witwatersrand, School of Molecular and Cell Biology, Braamfontein, Johannesburg, 2000, South Africa

**Keywords:** Cassava brown streak viruses, Wild host plants, Mechanical inoculation, Alternative host, Mozambique

## Abstract

Cassava brown streak disease (CBSD) caused by *Cassava brown streak virus* (CBSV) and *Ugandan cassava brown streak virus* (UCBSV) is the main constraint to cassava (*Manihot esculenta* Crantz) production in Mozambique. Using RT-PCR to amplify partial coat protein nucleotide sequences, we detected for the first time the occurrence of CBSV in two non-cassava perennial wild plant species: *Zanha africana* (*Radlk.*) Exell. and *Trichodesma zeylanicum* (Burm.f.) R.Br*.,* that occur widely within and near cassava fields in Nampula, Zambezia, Niassa and Cabo Delgado provinces. In addition, we also detected CBSV and UCBSV in *Manihot carthaginensis* subsp*. glaziovii* (Müell-Arg.) Allem., a wild cassava relative. These findings were verified in biological assays through mechanical inoculation of CBSV to *T. zeylanicum*, albeit at low rates of infection. Phylogenetic analysis clustered the CBSV isolates from the non-cassava plant species with those from cultivated cassava, with high sequence homology among CBSV (91.0–99.6%) and with UCBSV (84–92%) isolates. These results provide definitive evidence of a wider host range for CBSV and UCBSV in Mozambique, indicating that these viruses are not restricted to cultivated cassava. Our findings are key to understanding the epidemiology of CBSD and will aid in the development of sustainable management strategies for the disease.

## Highlights

• Alternative host plants of Cassava brown streak viruses were determined.
• There was evidence of natural infection of CBSV in non-cassava relative plants.
• CBSV was successfully transmitted in *T. zeylanicum* by mechanical inoculation.
• CBSV and UCBSV, associated with CBSD in cassava, naturally infect *M. glaziovii*.

## 1 Introduction

Cassava (*Manihot esculenta* Crantz, family Euphorbiaceae) is the second most important crop after maize in Mozambique [1]. More than 80% of cassava production in Mozambique occurs in the north and central regions. Currently, production in these regions is severely constrained by two cassava brown streak viruses, *Cassava brown streak virus* (CBSV) and *Ugandan cassava brown streak virus* (UCBSV) [[2], [3], [4]], which cause Cassava brown streak disease (CBSD) [2,5]. The disease was first reported to be transmitted with very low efficiency by whitefly, *Bemisia tabaci* (Gennadius) [6,7], but [8] recently confirmed generally moderate rate of transmission of CBSV, ranging from 30 to 53% using 20 to 100 whiteflies. Recently, the presence of the DAG motif in CBSV sequences suggests that aphids could be potential vectors of CBSV as observed in Squash vein yellowing virus (SqVYV) and Coccinia mottle virus (COCMOV) [9]. Work to confirm aphid transmission of CBSVs is ongoing.

A virus disease survey of cassava was undertaken in 1999 in Zambezia and Nampula provinces, which are the main areas of production in Mozambique in which CBSD was identified for the first time in Mozambique. Disease incidences in some fields reached 80–100% and many of the main cassava cultivars were affected [10]. In subsequent country-wide surveys in 2010 and 2012, CBSD was found in Zambezia, Nampula and a third province, Cabo Delgado, all in northern Mozambique. The disease was highest in Zambezia (61.3% and 82.2%) and lowest in Cabo Delgado (23.6% and 35.1%) in 2010 and 2012, respectively. The local cultivars ‘Cadri’ and ‘Robero’ were the most affected, while ‘Likonde’ and ‘Amwalikampiche’ had low incidences and symptom severity, indicating some tolerance to the disease [11]. When compared to previous surveys conducted in 1999 and 2003, the increasing incidence and symptom severity suggests that farmers were replanting new fields with disease-affected cuttings. Recently, the 2015 and 2017 country-wide surveys indicated a reduction in CBSD incidence and severity, attributed mainly to wide adoption of improved cassava cultivars with increased tolerance to CBSD in Nampula and Zambezia (Nurbibi Cossa, unpublished and Cassava Disease Diagnostic annual reports).

The natural occurrence of Cassava brown streak viruses in *M. carthaginensis* subsp*. glaziovii* (Müell-Arg.) Allem. has been reported [12]. In addition, *Nicotiana tabacum*, *N. benthamiana*, *N. debneyi*, *N. rustica*, *N. glutinosa*, *N. hesperis*, *N. occidentalis*, *Datura stramonium*, *Petunia hybrida*, *Chenopodium quinoa* and *C. amaranticolor* were used as experimental hosts for CBSV [13,14]; [15]. Of these plant species, *N. debneyi* and *N. benthamiana* have proved the most useful for virus infection assays [3,4,16]. Pathogens can have highly variable host ranges: in natural conditions some infect only one or a few related species (i.e., specialist pathogens), but others can infect a wide range of hosts. For example, *Tobacco rattle virus* reportedly infects over 400 plant species belonging to 50 different families [17] and *Cucumber mosaic virus* infects 1200 plant species belonging to 100 families [18]. The Cassava mosaic begomoviruses (CMBs) that cause Cassava mosaic disease (CMD) naturally occur in cassava, but also infect *Jatropha curcas* under experimental and natural conditions [19]. [20] reported *African cassava mosaic virus* (ACMV) and *East African cassava mosaic virus* (EACMV) in *M. carthaginensis* subsp*. glaziovii* (Müell-Arg.) Allem., *Senna occidentalis* L. and the weed *Combretum confertum* Benth. Therefore, given these findings of alternative hosts for several crop-infecting viruses, including some important in cassava, it is plausible that CBSV or UCBSV could have additional, yet undiscovered alternative hosts. There is limited information on alternative hosts and their potential role in the spread of CBSV and UCBSV in sub-Saharan Africa. The lack of knowledge of the alternative hosts of CBSV and UCBSV is a key knowledge gap in the epidemiology and management of CBSD, especially in the endemic countries such as Mozambique. Available information on the natural host range of Cassava brown streak viruses indicates that they are largely restricted to cassava and wild relatives such as *M. carthaginensis* subsp*. glaziovii* (Müell-Arg.) Allem. This study aimed to identify alternative host plants for Cassava brown streak viruses in Mozambique.

## 2 Materials and methods

### 2.1 Areas surveyed and sample collection

To determine and identify alternative hosts for CBSV, leaf samples were collected in 2014 from four major cassava production areas namely Nampula, Zambezia, Niassa and Cabo Delgado. A total of 120 leaf samples showing virus-like disease symptoms such as chlorosis, yellow spotting, deformation, mosaic, wilting, leaf curling and necrotic lesions were collected from 15 plant species: *M. carthaginensis* subsp*. glaziovii* (Müell-Arg.) Allem., *Mucuna pruriens*, *Cajanus cajan* (L.) Millsp., *Trichodesma zeylanicum* (Burm.f.) R.Br., *Paederia bojeriana* (A.Rich.) Drake subsp. *foetens* (Hiern), *Commelina benghalensis*, *Ageratum conyzoides* (L.), *Vernonia petersii* Oliv. & Hiern ex Oliv., *Zanha africana* (Radlk.) Exell., *Brachistegia spiciform* Benth, *Ocimum africanum* Lour., *Senna obtusifolia* (L.) H.S.Irwin & Barneby, *Ipomea tenuipes* Verdc., *Vernonia cinerea* (L.) Less. and *Nidorela* sp. (Table 1) growing within or nearby (5–10 m away) cassava fields. The wild plant species were identified using a working list of all plant species website (http://www.theplantlist.org). Additionally, wild plant species collected in the fields were taken to the Botany Department at Mozambique Agricultural Research Institute for identification and further confirmation of the identity/taxonomy by a Botanist. The samples were labeled and kept in herbarium field kits to preserve their integrity until laboratory analysis.

**Table 1:** Occurrence and ecology of non-cassava plant species sampled for Cassava brown streak viruses in Mozambique 2014.

Local name	Botanical name	Plant family	CBSV/UCBSV Testing results	Disease severity (1–5 scale)	Collection environment and ecology	Sample location	Frequency occurrence and distribution
Tree cassava	*Manihot carthaginensis* subsp*. glaziovii* (Müell-Arg.) Allem.	Euphorbiaceae (perennial shrub tree)	CBSV and UCBSV	4	Nearby cassava fields along boundaries and homestead	Zambezia, Nampula, Niassa	High
Velvet-fruited zanha	*Zanha africana* (Radlk.) Exell.	Sapindaceae (perennial shrub tree)	CBSV	3	Within cassava fields and uncultivated areas	Nampula	High
Camel bush	*Trichodesma zeylanicum* (Burm.f.) R.Br.	Boraginaceae (annual/perennial weed)	CBSV	3	Within cassava fields and uncultivated areas	Nampula, Niassa	Very high
Velvet bean	*Mucuna pruriens*	Fabaceae (creeping vine legume)	–	4	Within cassava fields and uncultivated areas	Nampula	Low
Pigeon pea	*Cajanus cajan* (L.) Millsp.	Fabaceae (annual/perennial legume)	–	2	In the cassava fields	Zambezia	High
Paederia bojeriana	*Paederia bojeriana* (A.Rich.) Drake subsp. *foetens* (Hiern)	Rubiaceae	–	3	In cassava fields and within cassava fields	Zambezia	Low
Benghal dayflower	*Commelina benghalensis*	Commelinaceae (annual/perennial herb)	–	2	In cassava fields and within cassava fields	Nampula	High
Billygoat weed	*Ageratum conyzoides* (L.)	Asteraceae (perennial weed)	–	3	In cassava fields and within cassava fields	Zambezia, Niassa, C.Delgado	High
–	*Vernonia petersii* Oliv. & Hiern ex Oliv.	Compositae (annual weed)	–	3	Within cassava fields and uncultivated areas	Zambezia, Nampula	Low
Zebrawood or Msasa	*Brachistegia spiciform* Benth	Fabaceae (perennial shrub tree)	–	3	Within cassava fields and uncultivated areas	Nampula	Very high
Lemon basil	*Ocimum africanum* Lour.	Lamiaceae (annual weed)	–	4	Nearby cassava fields and in the cassava field	Nampula	Very high
Cofeeweed/cassia	*Senna obtusifolia* (L.) H.S.Irwin & Barneby	Caesalpinioideae (annual/perennial herb)	–	3	Nearby cassava fields and in the cassava field	Nampula, Zambezia	Very high
Morning glory	*Ipomea tenuipes* Verdc.	Convolvulaceae (perennial)	–	2	Nearby cassava fields	Zambezia, Cabo Delgado	Low
Dandotapala	*Vernonia cinerea* (L.) Less	Asteraceae (annual shrub)	–	3	Within cassava fields and uncultivated areas	Zambezia, Nampula	Low
–	*Nidorela* sp.		–	2	Nearby cassava fields	Niassa	Low

### 2.2 CBSD symptoms severity

To score the CBSD symptoms severity in *M. carthaginensis* subsp. *glaziovii* (Müell-Arg.), we used more comprehensive descriptions based on 1–5 scale of foliar CBSD symptom described by Refs. [21] and [22]: 1 = no visible symptoms, 2 = mild vein yellowing or chlorotic blotches on some leaves, 3 = pronounced/extensive vein yellowing or chlorotic blotches on leaves, but no lesions or streaks on stems, 4 = pronounced/extensive vein yellowing or chlorotic blotches on leaves and mild lesions or streaks on stems, and 5 = pronounced/extensive vein yellowing or chlorotic blotches on leaves and severe lesions or streaks on stems, defoliation and dieback.

### 2.3 RNA extraction

Total RNA was extracted from the leaf samples using a modified CTAB protocol as described previously [23,24]. The yield of RNA was quantified using a Thermo Scientific NanoDrop 2000/2000c (Thermo Scientific, Waltham, MA, USA) (full spectrum UV–Vis) at A260/280 ratio.

### 2.4 Reverse transcription

Total RNA (4 μg) was used to synthesize cDNA in two steps using an ImProm-II™ reverse transcriptase Kit (Promega, Madison, WI, USA) following the manufacturer's instructions. RT was performed with cycling conditions of 42 °C for 60 min and 70 °C for 10 min and the resulting cDNA was used for PCR.

### 2.5 PCR amplification

To screen for the presence of CBSV in the samples, PCR was conducted using the primers CBSDDF and CBSDDR, which are designed to amplify the partial coat protein (CP) gene and 3′-untranslated region (UTR) [12] – with expected fragment sizes of 344 bp (CBSV) and 430–440 bp (UCBSV). The PCR reaction mix of 25 μL consisted of 12.9 μL of sterile de-ionized water, 3.0 μL of 10 × PCR buffer +20 mM MgCl_2_, 1.0 μL of primers CBSDDF/CBSDDR (10 mM), 0.3 μL of *Pfu* DNA polymerase, 2.8 μL of dNTPs (2.5 mM) and 4.0 μL of cDNA template. The PCR cycling conditions were 94 °C for 2 min, followed by 35 cycles of 94 °C for 30 s, 51 °C for 30 s and 72 °C for 30 s for denaturation, annealing and extension, respectively. PCR products were analyzed by electrophoresis in 1 × TAE buffer on a 2% agarose gel stained with 0.5 μg/mL of ethidium bromide.

### 2.6 Cloning and sequencing

Samples with the expected product size (344 bp for CBSV and 440 bp UCBSV) from PCR were cloned separately using a Thermo Scientific CloneJET PCR Cloning Kit and transformed into *E. coli* JM109 (Thermo Scientific), following the manufacturer's instructions. Samples with two amplified bands were cut from the gel and purified using a GeneJET Gel Extraction Kit (Thermo Scientific) following the manufacturer's instructions and cloned as for the samples with one band. Recombinant DNA was extracted using a GeneJET Plasmid Miniprep Kit (Thermo Scientific), and sent for sequencing by Inqaba Biotech (Pretoria, South Africa).

### 2.7 Phylogenetic analysis of CBSV sequences

The resulting sequences were trimmed and edited using FinchTV 1.4.0 (http://jblseqdat.bioc.cam.ac.uk/gnmweb/download/soft/FinchTV_1.4/doc/) and multiple alignments representing partial CP and 3′-UTR sequences were performed using MEGA 5.02. Nucleotide sequences of isolates obtained from cassava relatives and non-relatives were aligned and compared with all available GenBank CBSV and UCBSV sequences from eastern and southern Africa as well as CBSV sequences from cassava collected in Mozambique during this study. Phylogenetic analysis was performed using the maximum likelihood method as implemented in MEGA 5.02 [25]. All phylogenetic analyses were performed using the best-fit substitution model for nucleotides (GTR + I + G) with 1000 bootstrap replicates.

### 2.8 Mechanical transmission of CBSV

#### 2.8.1 Establishment of test plants

Infection assays of CBSV were established using *T. zeylanicum*, which was easier to grow than the shrub tree *Z. africana.* The plants were raised using seeds established in Hygromix growth medium (Hygrotech Pty Ltd, South Africa) and maintained under natural light in a screen house. Cypermethrin insecticide was applied weekly to the plants to control infestation by insects and possible transmission of viruses, and the plants maintained in an insect-proof net cage until inoculation.

#### 2.8.2 Virus sources and mechanical transmission

**Fig. 1:**
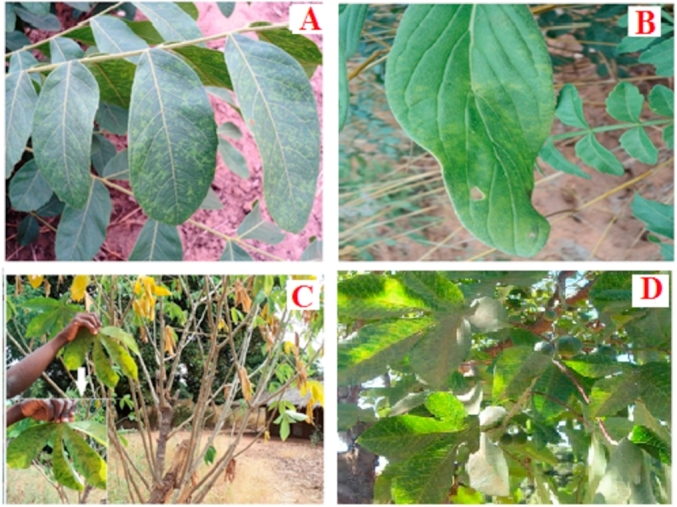
Array of viral disease symptoms on wild non-cassava plant species detected with Cassava brown streak viruses: (A) spotted yellowing along secondary veins, feathery chlorosis and yellow mosaic on leaves of Zanha africana (Radlk.) Exell, (B) yellowing, feathery chlorosis and leaf curling on leaves of *Trichodesma zeylanicum* (Burm.f.) R.Br. and (C & D) chlorosis and yellowing on leaves of Manihot carthaginensis subsp. glaziovii (Müell-Arg.) Allem, in Mozambique, 2014.

A bioassay experiment for CBSV transmission was conducted using classical virology methods for mechanical inoculation as described by Refs. [26] and [27]. Thirty plants of *T. zeylanicum* were used for the infection assays, among which five were included as controls. Extracts of CBSD-symptomatic cassava leaves confirmed to be positive for CBSV in RT-PCR (Fig. 1A) were used as sources of virus inoculum and were rubbed onto the expanded leaf surfaces of 25 T*. zeylanicum* plants with aid of carborundum dust (Fig. 1B). For negative control plants, only buffer (0.02 M Phosphate, PH = 7.0) was applied to the leaves. The inoculated plants were covered with transparent plastic and maintained in a controlled environment in the laboratory for 48 h at 25 °C. The plants were transferred to the greenhouse where they were monitored for symptom development. Plants were inspected daily for symptom development for one month, and the leaves tested for the presence of Cassava brown streak viruses using RT-PCR.

## 3 Results

### 3.1 Viral disease symptoms on alternative host plants

Viral disease symptoms on Velvet-fruited zanha (*Z. africana* (Radlk.) Exell) and Camel bush (*T. zeylanicum* (Burm.f.) R.Br.) included: spotted yellowing along secondary veins, feathery chlorosis, yellow mosaic and leaf curling (Fig. 1A and B). In comparison, the cassava relative *M. carthaginensis* subsp. *glaziovii* (Müell-Arg.) Allem had typical severe chlorosis with severity scale of 4, on the 1–5 severity scale described by Refs. [21] and [22] on the leaves and necrosis on the stems (Fig. 1C and D). The symptoms were similar to those observed on cultivated cassava. The incidence of plants with virus-like disease symptoms was moderate (45–55%) to high (80–90%) in the study locations (data not provided), and this formed the basis for sampling the plant species reported here.

### 3.2 PCR amplification of Cassava brown streak viruses in non-cassava plants

A total of 120 plant samples comprising of weeds, shrubs, trees and cassava relatives were screened for presence of CBSV and UCBSV using species-specific primers. PCR analysis produced the expected bands of 344 bp and 440 bp for CBSV and UCBSV, respectively. CBSV was detected in six plant samples: four of cassava relative *M. carthaginensis* subsp*. glaziovii* (Müell-Arg.) Allem. and two non-cassava plant species, *T. zeylanicum* (Burm.f.) R.Br. and *Z. africana* (Radlk.) Exell. UCBSV was detected in one *M. carthaginensis* subsp*. glaziovii* (Müell-Arg.) Allem. sample. The rest of the samples that did not test positive with Cassava brown streak viruses were kept for future study to determine the causal viruses for the virus-like symptoms and establish their importance to agriculture.

### 3.3 Phylogenetic analysis

**Fig. 2:**
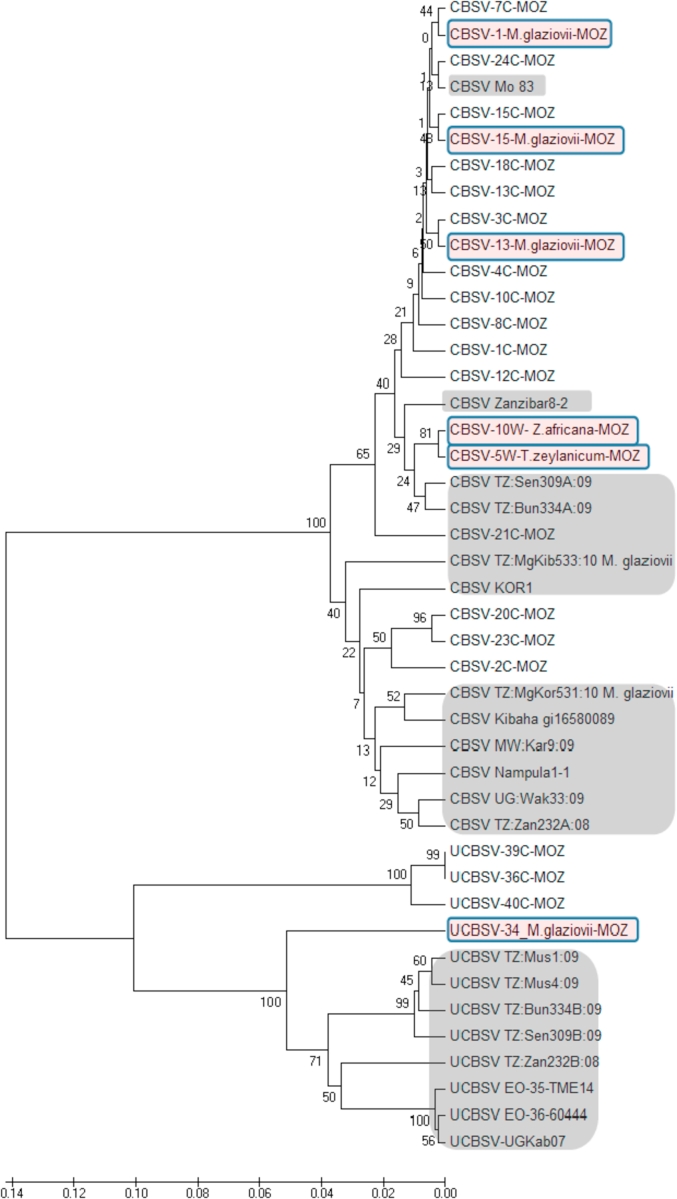
Phylogenetic tree constructed using the neighbor-joining method with MEGA5.2. The phylogenetic tree was generated based on partial CP-encoding nucleotide sequences of CBSV and UCBSV isolates collected in Nampula, Zambezia and Niassa Provinces. CBSV and UCBSV sequences from cassava relatives and non-relatives are indicated with pink shading, the reference isolates from GenBank are indicated with gray and the remaining are sequences from isolates collected during this study from cultivated cassava plants in Mozambique (isolates with terminal MOZ). The number at each branch represents the bootstrap value (1000 replicates).

Phylogenetic analysis was carried out to determine the genetic relationships among the six CBSV isolates obtained from the non-cassava samples using partial sequences of the core region of CP and 3′-UTR. The partial sequences were aligned with 20 reference nucleotide sequences (11 of CBSV and eight of UCBSV) from GenBank (Table 2) using MEGA 5.02 [25] with a best-fit model. As expected, comparisons based on nucleotide sequences revealed the existence of two major groups: CBSV and UCBSV. Five out of six sequences clustered with CBSV sequences from Mozambique (Fig. 2), while one of the sequences obtained from *M. carthaginensis* subsp*. glaziovii* (Müell-Arg.) Allem. clustered with UCBSV (Fig. 2). Isolates obtained from *T. zeylanicum* (Burm.f.) R.Br., *Z. africana* (Radlk.) Exell and *M. carthaginensis* subsp*. glaziovii* (Müell-Arg.) Allem. shared 91.0–99.6% sequence similarity with CBSV affecting cassava in East Africa and Mozambique. However, the UCBSV isolate from *M. carthaginensis* subsp*. glaziovii* (Müell-Arg.) Allem. had lower sequence homology (84–92%) with isolates from cultivated cassava.

**Table 2:** Cassava brown streak viruses isolates sequences used in the phylogenetic analysis in this study.

Isolate name	Host	Accession number	Reference
UCBSV TZ:Mus1:09	*M. esculenta* Crantz	HM453037	[28]
UCBSV TZ:Mus4:09	*M. esculenta* Crantz	HM453038	[28]
UCBSV TZ:Sen309B:09	*M. esculenta* Crantz	HM453036	[28]
UCBSV EO-36-60444	*M. esculenta* Crantz	KJ606231	[29]
UCBSV-UGKab07	*M. esculenta* Crantz	HG965222	[28]
UCBSV TZ:Bun334B:09	*M. esculenta* Crantz	HM453039	[28]
UCBSV TZ:Zan232B:08	*M. esculenta* Crantz	HM453040	[28]
CBSV TZ:Sen309A:09	*M. esculenta* Crantz	HM453033	[28]
UCBSV EO-35-TME14	*M. esculenta* Crantz	KJ606230	[29]
CBSV Nampula1-1	*M. esculenta* Crantz	HM346953	[28]
CBSV TZ:MgKor531:10 M. glaziovii	*M. carthaginensis* subsp*. glaziovii* (Müell-Arg.)	HM453032	[12]
CBSV KOR1	*M. esculenta* Crantz	GU563327	[12]
CBSV Mo 83	*M. esculenta* Crantz	FN434436	[4]
CBSV MW:Kar9:09	*M. esculenta* Crantz	HM171296	[28]
CBSV UG:Wak33:09	*M. esculenta* Crantz	HM171312	[28]
CBSV TZ:MgKib533:10 M. glaziovii	*M. carthaginensis* subsp*. glaziovii* (Müell-Arg.)	HM453031	[12]
CBSV TZ:Zan232A:08	*M. esculenta* Crantz	GU563325	[12]
CBSV TZ:Bun334A:09	*M. esculenta* Crantz	HM450034	[28]
CBSV Zanzibar8-2	*M. esculenta* Crantz	HM346957	[28]
CBSV-10W-_Z.africana-MOZ	*Zanha africana*	Yet to be received	This study
CBSV-10C-MOZ	*M. esculenta* Crantz	Yet to be received	This study
CBSV-18C-MOZ	*M. esculenta* Crantz	Yet to be received	This study
CBSV-1C-MOZ	*M. esculenta* Crantz	Yet to be received	This study
CBSV-13C-MOZ	*M. esculenta* Crantz	Yet to be received	This study
CBSV-15C-MOZ	*M. esculenta* Crantz	Yet to be received	This study
CBSV-2C-MOZ	*M. esculenta* Crantz	Yet to be received	This study
CBSV-3C-MOZ	*M. esculenta* Crantz	Yet to be received	This study
CBSV-4C-MOZ	*M. esculenta* Crantz	Yet to be received	This study
CBSV-5W-T.zeylanicum-MOZ	*Trichodesma zeylanicum*	Yet to be received	This study
CBSV-7C-MOZ	*M. esculenta* Crantz	Yet to be received	This study
CBSV-8C-MOZ	*M. esculenta* Crantz	Yet to be received	This study
CBSV-12C-MOZ	*M. esculenta* Crantz	Yet to be received	This study
CBSV-20C-MOZ	*M. esculenta* Crantz	Yet to be received	This study
CBSV-21C-MOZ	*M. esculenta* Crantz	Yet to be received	This study
CBSV-23C-MOZ	*M. esculenta* Crantz	Yet to be received	This study
CBSV-24C-MOZ	*M. esculenta* Crantz	Yet to be received	This study
CBSV-13-Glaziovii-MOZ	*M. carthaginensis* subsp*. glaziovii* (Müell-Arg.)	Yet to be received	This study
CBSV-15-Glaziovii-MOZ	*M. carthaginensis* subsp*. glaziovii* (Müell-Arg.)	Yet to be received	This study
CBSV-1-Glaziovii-MOZ	*M. carthaginensis* subsp*. glaziovii* (Müell-Arg.)	Yet to be received	This study

### 3.4 Koch's postulates and virus infection assays

**Fig. 3:**
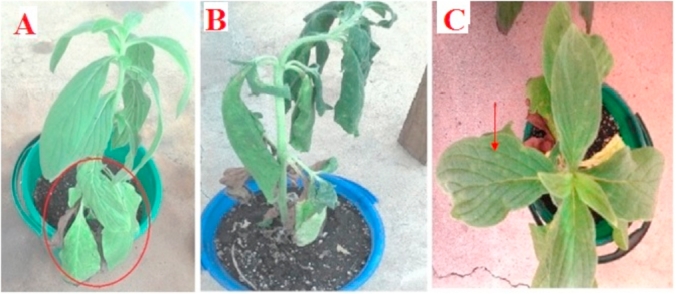
Symptoms induced by CBSV isolate (CBSV-8C-MOZ) after 5 weeks; 3 out of 25 inoculated *Trichodesma zeylanicum* (Burm.f.) R.Br. plants displayed viral disease symptoms, including (A) leaf yellowing, (B) wilting and (C) chlorotic spots.

Out of the 25 T*. zeylanicum* (Burm.f.) R.Br. plants mechanically inoculated with CBSV, only three successfully developed viral disease symptoms. The first symptoms were recorded at 32 days after inoculation. The symptoms included chlorotic spots, leaf yellowing and wilting (Fig. 3A–C), and were similar to those observed on *T. zeylanicum* (Burm.f.) R.Br. in the field, except for the wilting. The presence of CBSV in the infected plants was confirmed with RT-PCR.

### 3.5 Occurrence and distribution of the alternative host plants

**Fig. 4:**
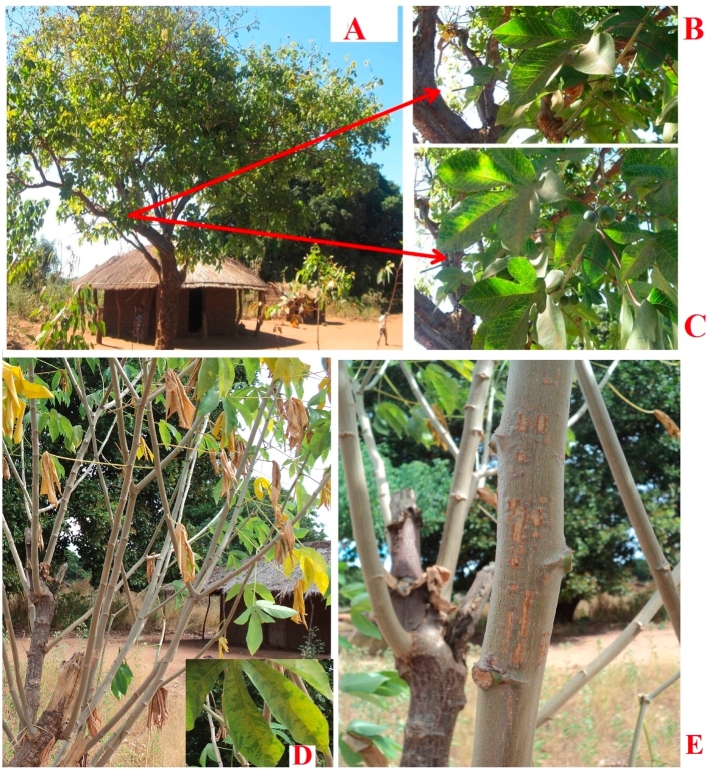
Occurrence of *Manihot carthaginensis* subsp. *glaziovii* (Müell-Arg.) Allem., plants in (A) homesteads, with typical CBSD symptoms on (B–D) leaves and (E) stems in the sampled areas in Mozambique, 2014.

**Fig. 5:**
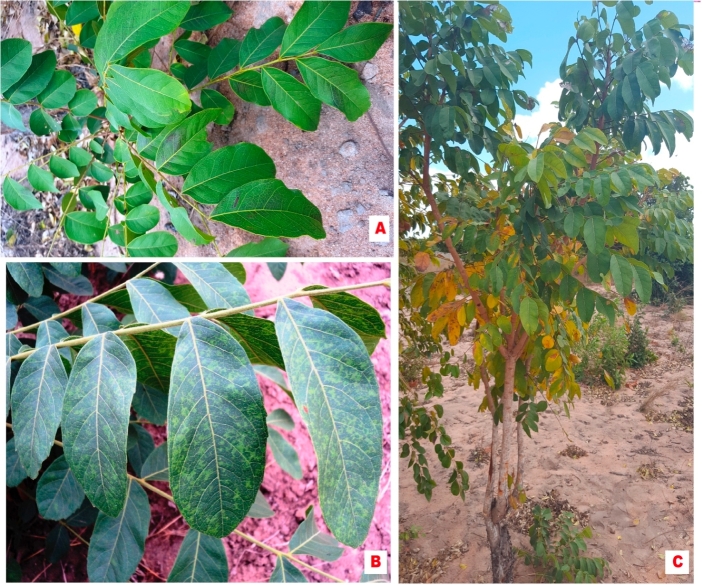
Symptomless (A) and viral disease symptomatic (B) plants of *Zanha africana* (Radlk.) Exell and (C) shrub/trees with viral disease symptoms growing in uncultivated areas next to cassava fields in the sampled areas in Mozambique, 2014.

**Fig. 6:**
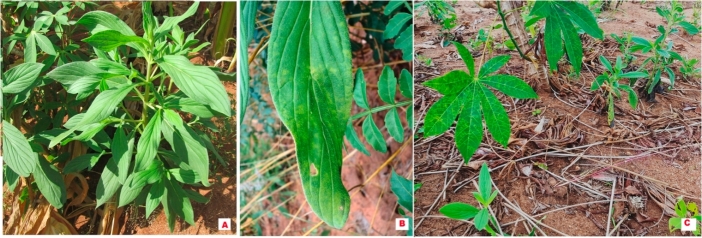
Occurrence of (A) symptomless and (B) viral disease symptomatic plants of *Trichodesma zeylanicum* (Burm.f.) R.Br. in a cassava field and (C) weed plants growing around cassava plants showing typical CBSD symptoms on leaves in Mozambique, 2014.

Occurrence and distribution of *M. carthaginensis* subsp. *glaziovii* (Müell-Arg.) Allem., the wild cassava relative and the two non-cassava plant species *T. zeylanicum* (Burm.f.) R.Br.and *Z. africana* (Radlk.) Exell in Nampula, Zambezia, Niassa and Cabo Delgado provinces were assessed in general terms as either low, high or very high. The *M. carthaginensis* subsp. *glaziovii* (Müell-Arg.) Allem. occurred with high frequency as shrubs along boundaries of the sampled cassava fields, in homesteads and in uncultivated areas (Fig. 4). *Zanha africana* (Radlk.) Exell plants occurred with low frequency as short shrubs and/or stumps within and near the sampled cassava fields. In uncultivated areas, *Z. africana* (Radlk.) Exell plants occurred with high frequency mainly as trees (Fig. 5). However, *T. zeylanicum* (Burm.f.) R.Br. plants were among the predominant weeds with very high frequency in cassava fields (Fig. 6). Due to their ease of growth through seed dispersal, this species is considered a major weed in agricultural fields (Table 1).

## 4 Discussion

We report here, for the first time, the occurrence of CBSV in two non-cassava perennial wild plant species, Velvet-fruited zanha (*Z. africana* (Radlk.) Exell) and Camel bush (*T. zeylanicum* (Burm.f.) R.Br.), and UCBSV in *M. carthaginensis* subsp*. glaziovii* (Müell-Arg.) Allem., a wild cassava relative in Mozambique, based on results obtained in PCR using virus species-specific primers [12] and phylogenetic analyses of the partial CP sequences of the isolates. Pairwise nucleotide sequence comparisons revealed high sequence homology among CBSVs (91.0–99.6%) and UCBSV (84–92%) isolates. The viral disease symptoms recorded on *Z. africana* (Radlk.) Exell and *T. zeylanicum* (Burm.f.) R.Br.) in the field included spotted yellowing along secondary veins, feathery chlorosis, yellow mosaic and leaf curling. In comparison, *M. carthaginensis* subsp*. glaziovii* (Müell-Arg.) Allem. had severe chlorosis on leaves and necrosis on stems, symptoms typical of CBSD on cultivated cassava. CBSV was detected in more samples, including *M. carthaginensis* subsp*. glaziovii* (Müell-Arg.) Allem., *Z. africana* (Radlk.) Exell and *T. zeylanicum* (Burm.f.) R.Br.), than UCBSV which occurred only in *M. carthaginensis* subsp*. glaziovii* (Müell-Arg.) Allem. A recent study by Ref. [31] reported CBSV to have a more rapid rate of evolution, and to be the predominant virus associated with severe CBSD compared with UCBSV in Uganda. In Mozambique, [11] showed that CBSV was widely distributed and the most important species causing CBSD. In contrast, this study observed that UCBSV was confined to Zambezia Province in *M. carthaginensis* subsp*. glaziovii* (Müell-Arg.) Allem, tree cassava, which is a glabrous shrub or tree that grows to 6 m high, and occasionally taller (10–20 m). This perennial plant was introduced to Africa as a plantation crop for rubber production in the 19th century and quickly established as common flora in uncultivated areas. In the study areas of Mozambique, tree cassava occurred mainly as a boundary plant along farms and homesteads and was abundant in uncultivated areas. In many homesteads, a few plants were maintained as sources of leafy vegetables, the majority bearing clear viral disease symptoms. *Zanha africana* (Radlk.) Exell is a perennial tropical African savanna tree [[32], [33], [34], [35]]. In the current study, *Z. africana* (Radlk.) Exell occurred as short shrubs and/or stumps in and near the sampled cassava fields. *Trichodesma zeylanicum* (Burm.f.) R.Br.) is an annual/perennial weed species that is abundant in agricultural and unused fields. It is highly competitive, a quick grower and covers many areas. Of the three wild non-cassava host plant species, *T. zeylanicum* (Burm.f.) R.Br.) was the most abundant in the sampled cassava farmers’ fields.

We tested infection assays of CBSV isolated from cassava plants to *T. zeylanicum* (Burm.f.) R.Br.) raised from seed, and ably demonstrated the mechanical transmission of the virus from cassava to a non-cassava plant species, albeit at low rates of infection. We do not know the reasons for the low infection rates, but mechanical transmission of plant viruses can be very delicate even between herbaceous hosts. For example, plants with high levels of phenolic compounds, such as *T. zeylanicum* (Burm.f.) R.Br., were found to have high antibacterial and antiphytoviral activities [36,37], which inhibit disease development through inhibition of extracellular enzymes and antioxidant activity in plant tissue [38]. Similarly, resistance to mechanical viral infection in chili was attributed to increased quantity of phenolics [39]. Regarding transmission of cassava brown streak viruses [40], indicated that mechanical transmission could not be achieved by using a simple buffer in infection assays, and suggested the use of antioxidants in buffers to enhance mechanical inoculation. We suggest that future investigations could include grafting and/or vector mediated transmission in infection assays. However, notwithstanding the low infection rates in our study, mechanical transmission successfully confirmed *T. zeylanicum* (Burm.f.) R.Br. as a natural host for CBSV. Interestingly, the incidence of *M. carthaginensis* subsp*. glaziovii* (Müell-Arg.) Allem., *Z. Africana* (Radlk.) Exell and *T. zeylanicum* (Burm.f.) R.Br.) plants with viral disease symptoms that tested positive for CBSVs was moderate to high in the sampled areas. In this study, we did not investigate the vectors associated with transmission of the Cassava brown streak viruses detected in the non-cassava plant species and suggest this to be a focus for future research.

The high abundance and widespread distribution of *M. carthaginensis* subsp*. glaziovii* (Müell-Arg.) Allem., *Z. africana* (Radlk.) Exell and *T. zeylanicum* (Burm.f.) R.Br. plants in the CBSD-affected areas in Nampula and Zambezia suggests that these plants serve as important inoculum sources for Cassava brown streak viruses that infect cassava crops both during the season and off-season. We propose that a survey be conducted to further establish the incidence of CBSV infections in the three wild host plant species described in this study. In addition, awareness campaigns should be carried out to educate farmers, agricultural extension officers, scientists (plant breeders, entomologists and virologists) and other cassava stakeholders on the importance of wild non-cassava plant hosts in the spread and management of CBSD. Emphasis should be placed on disease symptom identification, scouting and roguing of suspected plants in cassava fields. Attempts should be made to plant cassava crops away from uncultivated areas with suspected viral disease symptomatic weeds, shrubs and trees, including the three wild plant hosts identified in this study, although this may be a challenge to achieve in areas with limited arable land and/or a lack of community participation.

## Declaration of conflict of interest

The authors had no conflict of interest.
